# The conformation of enkephalin bound to its receptor: an “elusive goal” becoming reality

**DOI:** 10.3389/fmolb.2014.00014

**Published:** 2014-10-07

**Authors:** Domenico Sanfelice, Piero A. Temussi

**Affiliations:** ^1^Molecular Structure, MRC National Institute for Medical ResearchLondon, UK; ^2^Dipartimento di Chimica, Universita' di Napoli Federico IINapoli, Italy

**Keywords:** peptides, opioids, receptors, NMR, docking

## Abstract

The availability of solid state structures of opioid receptors has prompted us to reconsider a crucial question concerning bioactive peptides: can their conformation be studied without any knowledge of the structure of their receptors? The possibility of giving a meaningful answer to this query rests ultimately on the ease of dealing with the flexibility of bioactive peptides, and amongst them one of the most flexible bioactive peptides, enkephalin. All solution studies of enkephalin hint at an inextricable mixture of quasi isoenergetic conformers. In this study we refer to the only NMR work that yielded inter-residue NOEs, performed at very low temperature. In the present work, we have used the simplest possible docking methods to check the consistency of the main conformers of enkephalin with the steric requirements of the active site of the receptor, as provided by the crystal structure of its complex with naltrindole, a rigid antagonist. We show that the conformers found in the equilibrium mixture at low temperature are indeed compatible with a good fit to the receptor active site. The possible uncertainties linked to the different behavior of agonists and antagonists do not diminish the relevance of the finding.

## Introduction

The alkaloids of opium have been used for at least the last 4000 years to alleviate pain and for their euphoric effects. Enkephalins, the first endogenous opioids, are among the best studied bioactive peptides. They are pentapeptides found endogenously in all vertebrates (Simantov et al., 1976). In nature, they act by interacting with the same receptors that recognize plant opiates. The information is transmitted to the brain where it is processed to have effects both in terms of analgesia and drug dependence. Discovered in 1975 (Hughes et al., 1975), their sequences contain two aromatic residues in the first and fourth position and two consecutive glycines in position 2 and 3. A semi-conserved position 5 accommodates equally well leucine or methionine. Although their affinity for the μ receptor is lower than that of the most potent alkaloids, both enkephalins are better δ agonists than most alkaloids (Schiller et al., 1992). Because of their high biological significance and, at the same time, their relatively modest sequence length, enkephalins were also natural targets both for structural and theoretical studies attempting to define the free energy landscape of proteins (Purisima and Scheraga, 1987; Hansmann et al., 1999). Endless experimental studies both by X-ray crystallography and by NMR were dedicated to the attempt of capturing the “biologically active conformation” that is the conformation adopted by enkephalins when interacting with the receptor (reviewed in Spadaccini and Temussi, 2001). The main crystal structures of isolated enkephalins are still now exhaustively reviewed in a classical analysis (Deschamps et al., 1996). We adopted a different approach in solution studies.

Solution studies have shown that enkephalin is very flexible and exists in most media as a complex mixture of quasi isoenergetic conformations (Spadaccini and Temussi, 2001). Unraveling this mixture in terms of a few conformers is extremely difficult, even by sophisticated calculation approaches based on systematic exploration of the free energy landscape (Meirovitch et al., 1995; Meirovitch and Meirovitch, 1996). In the past, we proposed a “reverse” strategy which used rigid molecular molds as suitable filters for conformational searches (Amodeo et al., 1998).

The straightforward strategy generally followed in most solution studies of small bioactive peptides (e.g., see Temussi et al., 1989; Crescenzi et al., 1996) implies as a first step the determination of the most energetically favorable peptide conformers. These in turn are compared with rigid molds to check the consistency of the shape of the message domain. Finally, the conformers selected by this filter are used to improve the indirect mapping of the hypothetical receptor's active site. Owing to the extreme difficulty of finding a reasonable number of low energy conformers in the huge conformational space of enkephalin, in the past we adopted a reverse strategy (Amodeo et al., 1998). We used spectroscopic data only to check whether, even among simple conformational mixtures of the peptide, there are conformers consistent with the shape of rigid alkaloid opioids. Then we checked whether a mixture composed by a limited number of these conformers could account for NMR data and energy requirements. We showed that this is indeed possible by referring to a *naïf* equilibrium mixture, representative of the most common peptide turns. In such a mixture we found the presence of conformers of Leu-enkephalin consistent with the shape of δ selective rigid compounds that, at the same time, could account for high quality NOE data at low temperature (Amodeo et al., 1998).

This method allowed us to account for NMR data at low temperature using just a few enkephalin conformers (Amodeo et al., 1998). However, the identity of the receptor being unknown, these studies did not find an outcome related to the fit of the active site. As is true with many other biologically active linear peptides from natural sources, enkephalins are very flexible molecules that can assume a large number of conformations whose distribution is a function of the environment. Their physiologically relevant structure cannot easily be determined in the absence of their natural partners. In the past, there have been many attempts to circumvent these difficulties, particularly by choosing, in solution studies, environments as close as possible to natural media (Pastore and Temussi, 2013).

Despite some successes in the conformational analyses of enkephalins skepticism has persisted concerning the significance of these studies: does the bioactive conformation exist at all among the many conformations accessible to the receptor free peptide? This question is crucial also in view of the newly identified family of intrinsically unfolded proteins, that is, proteins which adopt a specific conformation only upon binding to their partners. Recently the structures of the three μ, δ, and κ opioid receptors were solved by crystallography (Granier et al., 2012; Manglik et al., 2012; Wu et al., 2012; Fenalti et al., 2014). The active sites of μ, δ, and κ receptors can be described as “exposed binding pockets,” similar in shape but differing in the residues lining the walls. This major breakthrough naturally opens the possibility of validating the results from previous conformational studies and, more interestingly, of understanding the molecular basis of the opioid-receptor interaction.

We can now show how availability of the structure of the δ selective opioid receptor and a rationally instructed docking approach leads us to find a receptor-bound conformation of enkephalin which is fully compatible with previous NMR studies. In particular, the two bioactive conformers described in this study are those found by the mentioned “reverse” strategy (Amodeo et al., 1998). Our studies have also important ramifications for studies of intrinsically unfolded proteins whose study is at present as trendy as that of “biologically active peptides” was a few decades ago (Pastore and Temussi, 2013) and their significance for defining crucial interactions with already folded proteins.

## Methods

Preliminary screening of a few hypothetical candidates of enkephalin bioactive conformation, coming from solid state studies or from a low temperature NMR study, was performed by superimposing the tyrosine aromatic ring of enkephalin conformers with the corresponding ring of Naltrindole. Superpositions were made using MOLMOL (Koradi et al., 1996).

### Virtual screening

The crystal structure of the Human δ opioid 7TM receptor was obtained from the Protein Data Bank (PDB) (id: 4N6H) (Fenalti et al., 2014) and edited for docking calculations using AutoDockTools 1.5.6 software: Na^+^ ion, naltrindole and water molecules were removed, polar hydrogens and partial charges were added explicitly, whereas the program automatically adds other hydrogens.

The modified structure of the human δ opioid receptor was used as a model for the docking procedure with software AutoDock Vina 1.2 (Trott and Olson, 2010). The docking protocol was initially set to rigid condition with a size of the dock grid of 24 × 24 × 28 Å, which encompasses the binding site for naltrindole. Exhaustiveness was initially set to 10 with all other parameters set on default values, then was increased to 20 for final dockings. Molecular models of an independently built naltrindole and of two conformers from NMR calculations (Amodeo et al., 1998) were used for virtual screening. All calculations were done on a 64 GPU Dell Cluster using 128 GB of RAM. The top-ranked complexes, sorted by binding energy values, were visually inspected for good stereochemical geometry and docking. For visualization, docking poses generated by AutoDock Vina were directly loaded into PyMol (http://www.pymol.org) through PyMOL Autodock/Vina Plugin (Seelier and de Bert, 2010). Images of the modeled receptor-ligands complexes were produced by PyMol and AutoDockTools 1.5.6.

### Docking energy analysis

Evaluation of the docking results was done using DrugScore Xtented (DSX) (Neudert and Klebe, 2011): the software estimates the binding energy of the pose of the ligand bound to the delta-opioid binding site by using a knowledge-based scoring function. DSX-score uses statistical pair potentials derived from Cambridge Structural Database (CSD) (Velec et al., 2005) and from PDB. Moreover, associated to PDB potential, Solvent Accessible Surface potential (SAS-potential) is introduced in DSX-score in order to account for desolvation effects. PDB and SAS potentials were used in this work. The ligand with the larger negative score has a theoretical higher affinity.

The two models for the final complexes of conformers A and B have been deposited in the PMDB database (http://bioinformatics.cineca.it/PMDB) with accession numbers PM0079713 and PM0079714.

## Results

### Choice of hypothetical bioactive conformers

**Figure 1:**
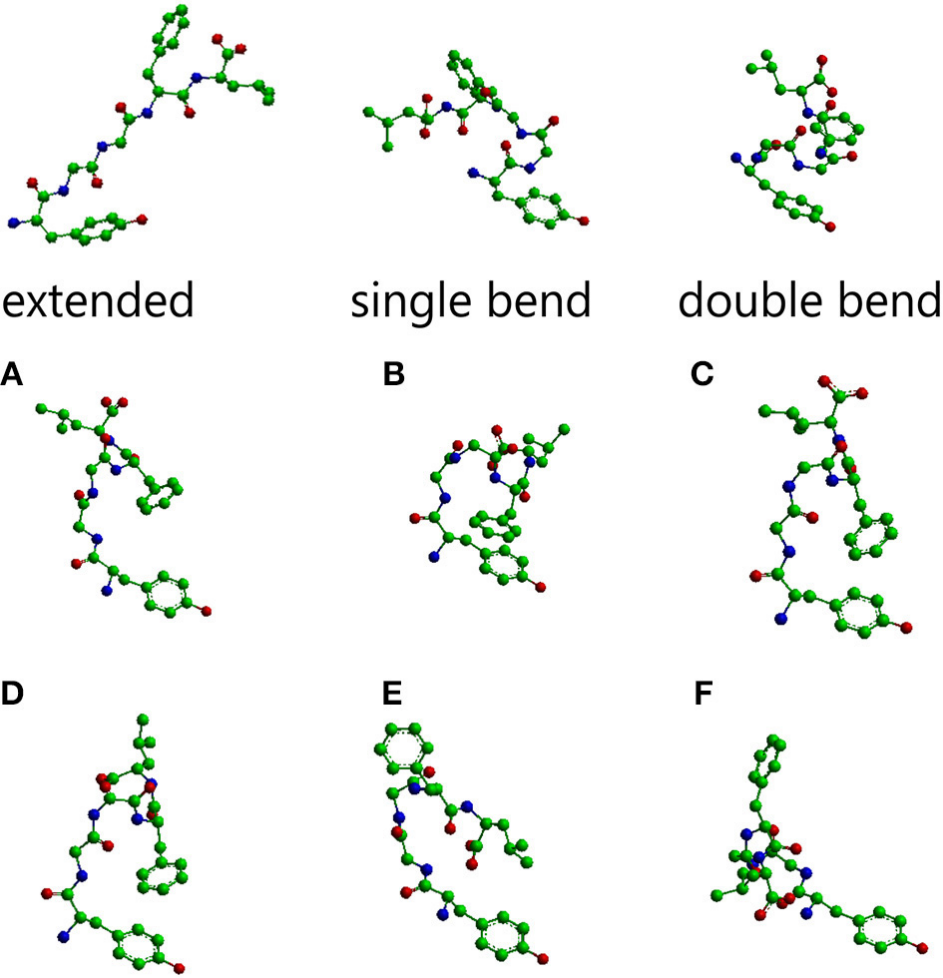
**Molecular models of Leu-enkephalin in the three main conformations (extended, single bend, double bend) found in solid state determinations of simple opioids (Deschamps et al., 1996) and the six main conformations **(A–F)** found in solution when the peptide is dissolved in a DMSO/water cryomixture at 275 K (Amodeo et al., 1998)**. All models are shown as stick and balls with carbon atoms as green balls, nitrogen atoms as blue balls, and oxygen atoms as red balls. Models were generated with Chem3D (trial version 11).

The main conformations of enkephalin found in crystals (Deschamps et al., 1996) have been classified in three categories, described as “extended,” “single bend,” and “double bend.” The conformers shown in Figure 1 are characterized by the values of ϕ/ψ angles reported in Table 1S (Supplementary Material), representing averages of the values reported in Table IIa of Deschamps et al. (1996) for each of the X-ray structures representative of “extended,” “single bend,” and “double bend” conformations.

As discussed in the introduction, aqueous solutions of enkephalin contain entangled mixtures of a very large number of quasi isoenergetic conformations. To disentangle putative bioactive conformers among these conformations we have proposed a so-called reverse strategy (Amodeo et al., 1998). The δ selective rigid compounds used as conformational sieves were 7-spiroindanyloxymorphone (SIOM), the first selective non-peptide δ_1_ opioid agonist (Portoghese et al., 1993) and BW373U86, a δ opioid agonist (Chang et al., 1993), novel at the time, with a piperazinyl-diphenylmethane skeleton characterized by a μ/δ selectively comparable to that of DPDPE (Calderon et al., 1994).

The conformations selected using the conformational sieves are characterized by the values of ϕ/ψ angles reported in Table 2S (Supplementary Material) (Amodeo et al., 1998). A suitable mixture of only two of these conformers (A and B) could account for experimental NOEs. It is obvious that experimental NOEs represent average values for all rapidly interconverting conformers in the distribution. For the sake of completeness, four additional conformers (C, D, E, F) were also considered as part of a very simple equilibrium mixture because, albeit not necessary to account for the NOEs, they are of comparable energy with respect to A, B. The molecular models of all these solution conformers, whose mixture can account for NMR data, are shown in Figure 1 alongside the solid state conformers.

### Inside the active site

Granier et al. (2012) have characterized in detail the active site of the δ-OR-T4L, which can be described as an exposed binding pocket, similar in shape to those previously observed for two other opioid receptors. A peptide as conformationally flexible as enkephalin can, in principle, adopt the ideal conformation to suit the receptor requirements even after entering the cavity: a paradigmatic case of induced fit. However, this induced fit might be slow if the starting conformation clashes severely with the walls of the cavity. It would be advantageous for the fit to deal with preselected conformers with a shape closer to the bioactive conformation. We decided to explore the compatibility of the enkephalin conformations described above with the shape of the binding pocket.

The receptor used for this comparison is the human δ receptor recently solved by Fenalti et al. (2014). The first step was to superimpose the tyrosine ring of enkephalin (in a given conformation) with the corresponding ring of the naltrindole message domain. The examples concerning the solid state conformations are shown in Figure 1S of Supplementary Material. None of the solid state conformers displayed in Figure 1S can be directly accommodated in the receptor without clashes. In particular, the extended conformation invades completely helix V of the receptor, the single bend goes across helix VII and the double bend conformer invades helix V. In addition, all models have the aromatic rings of Tyr^1^ and Phe^4^ wide apart in space, a feature at variance with the shape of naltrindole and/or the shape of δ selective agonists like SIOM (Portoghese et al., 1993) or BW373U86 (Chang et al., 1993). On the contrary, as shown in Figure 2S of Supplementary Material, some of the conformers found in the solution study look more promising. Although none of them fits into the pocket of the active site without clashes, most of them have the aromatic rings of Tyr^1^ and Phe^4^ in a relative spatial position consistent with that of δ selective agonists. In addition, most of them suffer from a comparatively less dramatic number of clashes with respect to the solid state conformers. Altogether, compact conformers look more promising than extended ones, in particular conformers A and B (Amodeo et al., 1998) have fewer clahes but, even more significantly, account for NMR NOEs observed at low temperature (Amodeo et al., 1998).

**Figure 2:**
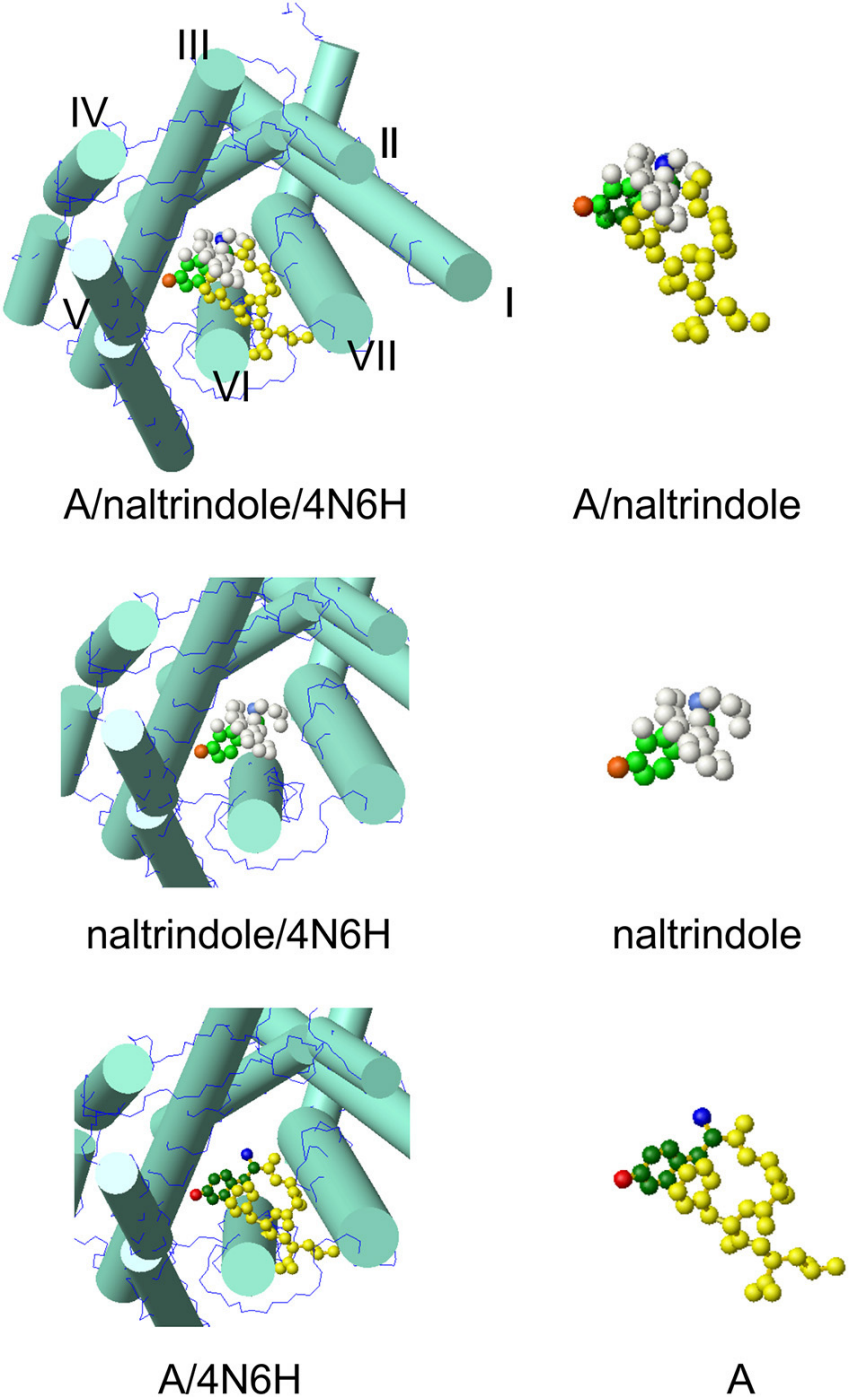
**Molecular model of conformer A of Leu-enkephalin found in solution (Amodeo et al., 1998) inside the active site of the receptor (pdb id 4N6H)**. The orientation is the result of the superposition of heavy atoms of the tyrosine ring to the corresponding atoms of naltrindole. Both enkephalin and naltrindole are shown as atom models. The carbon atoms of the tyrosine moiety are colored in green (dark green for enkephalin and pale green for naltrindole); the positive nitrogen atom is colored dark blue in enkephalin and pale blue in naltrindole; the oxygen of the tyrosine ring is dark red in enkephalin and pale red in naltrindole. Remaining atoms are all yellow in enkephalin and white in naltrindole. The receptor is shown schematically by drawing the main trans-membrane helices as cylinders (I–VII). All models were generated by MOLMOL (Koradi et al., 1996).

The model at the top of Figure 2 hosts the A conformer of enkephalin superimposed to the molecule of naltrindole inside the active site of the δ receptor. Superposition was achieved using the tyrosine aromatic ring and the corresponding aromatic ring of naltrindole. The two models in the middle and at the bottom show partial views of the receptor site hosting naltrindole and enkephalin, respectively. The molecular models on the right are those of naltrindole and Leu-enkephalin (A), either superimposed (top) or separated in the same orientation as seen inside the receptor. The models were generated with MOLMOL (Koradi et al., 1996).

On visual inspection, the best conformer, as can be appreciated from the pictures of Figure 2S and of Figure 2, is conformer A. It has only a few clashes with helix 6 of the receptor and has a good overall similarity with the shape of naltrindole, because of the close proximity of the aromatic rings of Tyr^1^ and Phe^4^. The second best is conformer B. Therefore, we have further checked whether the interaction with the receptor walls could be optimized by small adjustments of the molecular models of these two conformers and/or changes of the side chain conformations of relevant residues of the receptor site.

### Docking

All conformers chosen as paradigmatic examples from conformational studies that preceded the unveiling of the structure of the receptor are not perfect fits and indeed it would be very surprising if they were. What needs to be done to fit them inside the receptor without significant clashes? Is it at all feasible to make them fully consistent with the receptor requirements with a minimum of readjustment? To answer this question we chose conformers A and B from the quoted solution study (Amodeo et al., 1998) and performed their docking with three different starting strategies.

#### Docking of naltrindole

To initially test the efficiency of the docking software (AutoDock Vina), and to define the appropriate docking grid, we tried to dock naltrindole in a receptor previously deprived of “its” naltrindole. Instead, we used for naltrindole a molecular model generated with ChemDraw (2008). The molecular model of naltrindole was prepared for docking using AutoDockTools, imposing the rigidity of all bonds. Calculations converged to a position of naltrindole virtually identical to that observed in the crystal (4N6H), with a very good value of the affinity, as defined by the main software or by an evaluation software (DSX). The virtual screening results, obtained from AutodockVina, summarized in Table 1, were sorted on the basis of their predicted binding free energies (column labeled “Affinity”). In Table 1, together with the values of affinity directly furnished by AutoDock Vina, are reported the corresponding scores obtained with DSX (column labeled “Score”), a software that analyzes the output of Autodock. DSX estimates the relative binding energy of the ligands in the various configurations in the opioid receptor. The energy reported is the total score including possible torsion and intramolecular contributions; Per Contact Score (PCS) is the score divided by the number of atom–atom interactions having any contribution in the final score (number of contacts within 6 Å).

**Table 1:** **Virtual screening and docking energy analysis**.

**AutoDock Vina**			**DSX**	
**Mol**	**Pose^a^**	**Affinity^b^ (Kcal/mol)**	**Score (Kcal/mol)^c^**	**PCS^d^**
Naltrindole	1	−13.6	−144.989	−0.226
	2	−11.0	−109.005	−0.218
A - rigid	1	24.1	77.423	0.068
B - rigid	1	30.1	124.149	0.135
A flex	1	−7.6	−161.644	−0.166
	2	−7.5	−154.517	−0.163
	3	−7.5	−148.151	−0.152
	4	−7.5	−147.154	−0.166
B flex	1	−7.9	−161.321	−0.170
	2	−7.4	−153.970	−0.164
	3	−7.4	−152.024	−0.152
	4	−7.3	−151.257	−0.169
A (flex receptor)	1	−11.1	−112.435	−0.198
	2	−11.0	−109.233	−0.194
	3	−10.8	−106.120	−0.201
	4	−10.6	−103.971	−0.187
B (flex receptor)	1	−10.6	−104.742	−0.191
	2	−10.4	−103.128	−0.187
	3	−10.2	−101.951	−0.188
	4	−10.0	−99.873	−0.175

*Poses obtained from AutoDock Vina are sorted on the basis of their predicted binding free energy (Affinity). The DSX energy (Score) is the total score including possible torsion and intramolecular contributions of the various configurations in the opioid receptor. Per Contact Score (PCS) is the score divided by the number of atom–atom interactions having any contribution in the final score (number of contacts within 6Ǻ)*.

a *Number of poses classified by energy levels provided by AutoDock Vina*.

b *Calculated Energy of interaction*.

c *Predicted Energy (Score) of interaction*.

d *PCS, Per Contacts Score*.

**Figure 3:**
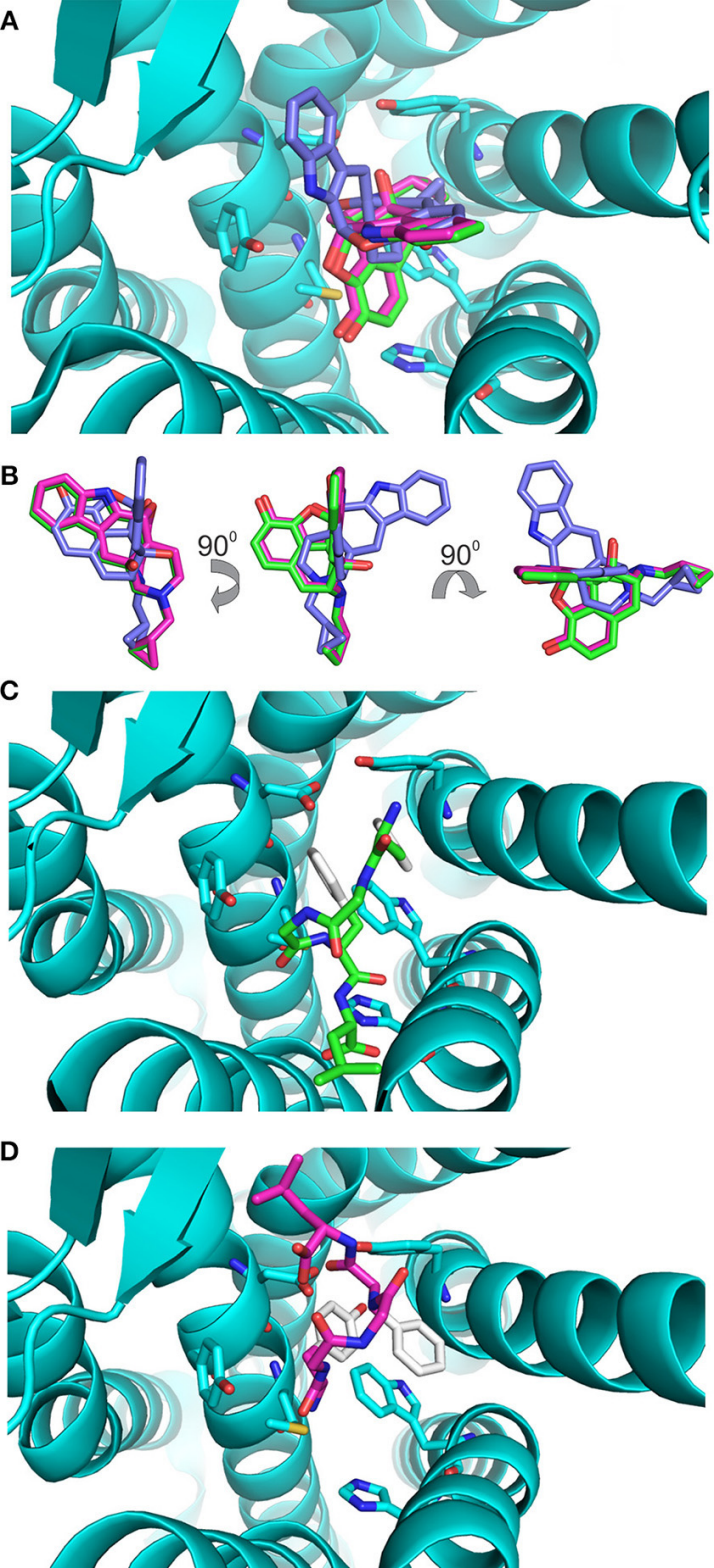
**Docking of the rigid ligands to the 4N6H protein. (A)** Molecular models of naltrindole docked on the rigid receptor. The molecular model found in the original crystal structure is shown as a stick model with carbon–carbon bonds colored in pink; the model corresponding to pose number 1 (green) coincides perfectly with the crystallographic one. The model corresponding to pose number 2 is shown with purple carbon–carbon bonds. **(B)** Relative orientation of the models of naltrindole corresponding to poses number 1 and 2. Rotations around vertical and horizontal axes are indicated to facilitate the recognition. **(C)** Final position of conformer A, docked as a rigid body inside the receptor cavity. The molecular model is shown as a stick model with carbon–carbon bonds colored in green, but for the aromatic rings of Y^1^ and F^4^, shown in white. The two aromatic rings are at the top of the cavity, far from the corresponding rings of naltrindole in the crystal structure. **(D)** Final position of conformer B, docked as a rigid body inside the receptor cavity. The molecular model is shown as a stick model with carbon–carbon bonds colored in dark pink, but for the aromatic rings of Y^1^ and F^4^, shown in white. The two aromatic rings are at the bottom of the cavity, roughly in the same portion of space with respect to the corresponding rings of naltrindole.

The values of these two parameters were then used to gauge the results with enkephalin conformers. Interestingly, the results of the docking of naltrindole produced two poses: the one with the lowest energy, pose 1, is identical to the crystallographic structure (see Figure 3A where the crystallographic molecule is colored in pink and the added one is shown in green), the second pose (the purple molecule of Figures 3A,B) has an energy comparable with that of pose one (see Table 1) and is tilted by approximately 90 degrees around a vertical axis passing through the cyclopropane ring (Figure 3B). The portion of space occupied inside the binding cavity by the tilted ligand is roughly the same but relevant moieties are oriented in a different way. Figure 3A, alongside the models of naltrindole, shows the sidechains of the residues closest to naltrindole (D128, Y129, N131, M132, W274, H278, Y308), chosen from the analysis of Ligplot+ (Figure 3S of Supplementary Material).

#### Docking of rigid conformers

Conformers A and B (see Figure 1) from the work of Amodeo et al. (1998) were chosen for further docking studies. Initially the two conformations of enkephalin were considered rigid in the virtual screening, i.e., assuming that no bonds are rotatable. As expected, the two conformations, although occupying a portion of space close to that of naltrindole, did not dock relevant moieties (aromatic rings and positive nitrogen) in the places found for naltrindole (Figures 3C,D) and led to poses of very high energy if compared with the reference value of naltrindole (see Table 1).

#### Docking of flexible enkephalin conformers

**Figure 4:**
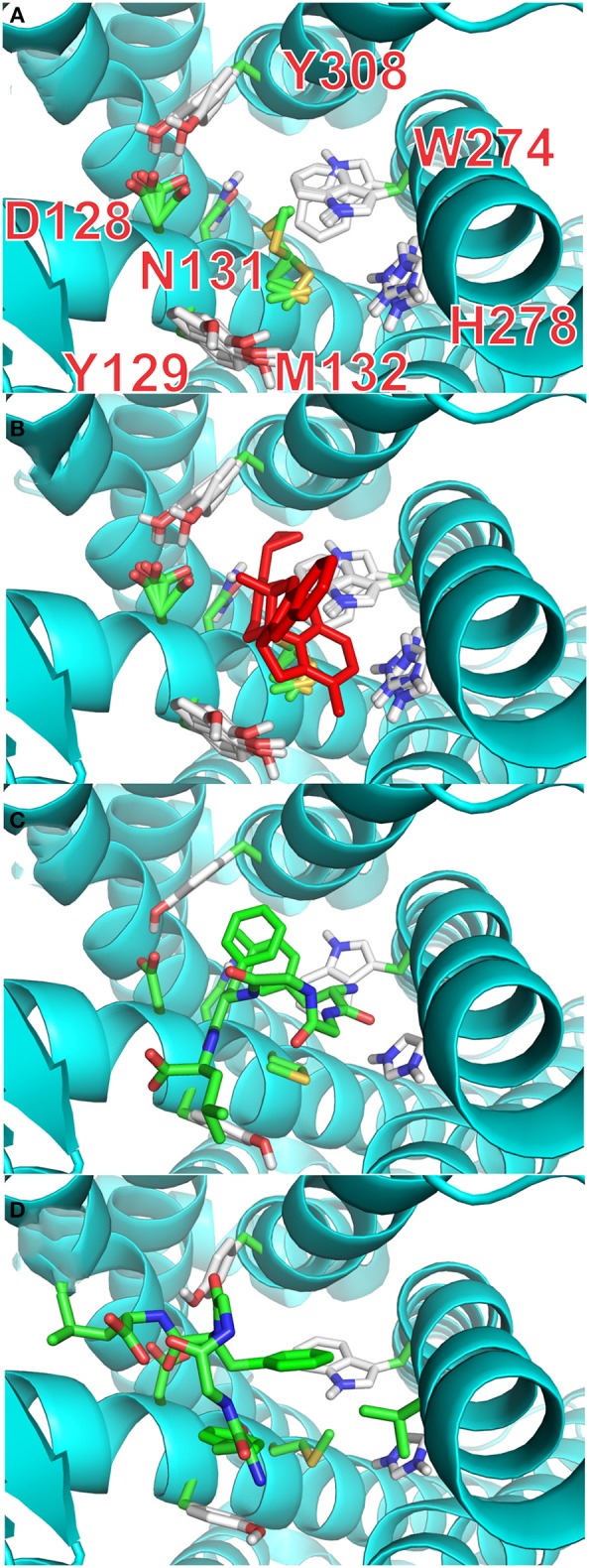
**Docking with flexible receptor for side chains. (A)** Level of flexibility for side-chains of the selected seven amino-acids is indicated by the superposition of their sidechains in the four lowest energy poses. Sidechains of seven critical residues of the active site of the receptor are represented as thin stick models and labeled with single letter notation: D128, Y129, N131, M132, W274, H278, and Y308. **(B)** Same view as **(A)** with the active site hosting the model of naltrindole in the original X-ray structure (red stick representation). **(C)** Lowest energy pose for the complex of rigid conformation A of enkephalin inside the active site of the receptor. The molecular model of conformer A is represented as sticks model in which the carbon–carbon bonds are colored green. **(D)** Lowest energy pose for the complex of rigid conformation B of enkephalin inside the active site of the receptor, shown together with the original naltrindole X-ray structure. The molecular model of conformer B is represented as sticks model in which the carbon–carbon bonds are colored green.

It is clear that docking rigid models of enkephalin in a rigid binding site has few chances of success. Our next step was to allow for almost complete flexibility of the peptide ligand, keeping the receptor rigid. Docking was started from conformations A and B. In both calculations nearly full flexibility was allowed, meaning that carbon–carbon bonds could rotate freely around the bond axis, while peptide bonds and aromatic rings were kept rigid. Both calculations lead to poses comparable in energy and geometry. Affinities and Scores are much lower than those obtained with rigid conformers but still higher than those of the naltrindole benchmark. It is possible to observe that the aromatic rings of enkephalin have a tendency to occupy the hydrophobic patches of the binding site. One of the poses for each starting conformer, i.e., A-4 and B-4 of Figure 4S (Supplementary Material), presents the aromatic rings in a relative orientation consistent with the geometry of naltrindole. The torsion angles of the two conformers diverge from those of the starting conformations but are not the same and cannot be classified in terms of canonical bends. This result is the umpteenth indication of the extreme flexibility of enkephalin. Figure 4S and Table 1 summarize the results for four of the lower energy results.

#### Docking with flexible receptor's side chains

**Figure 5:**
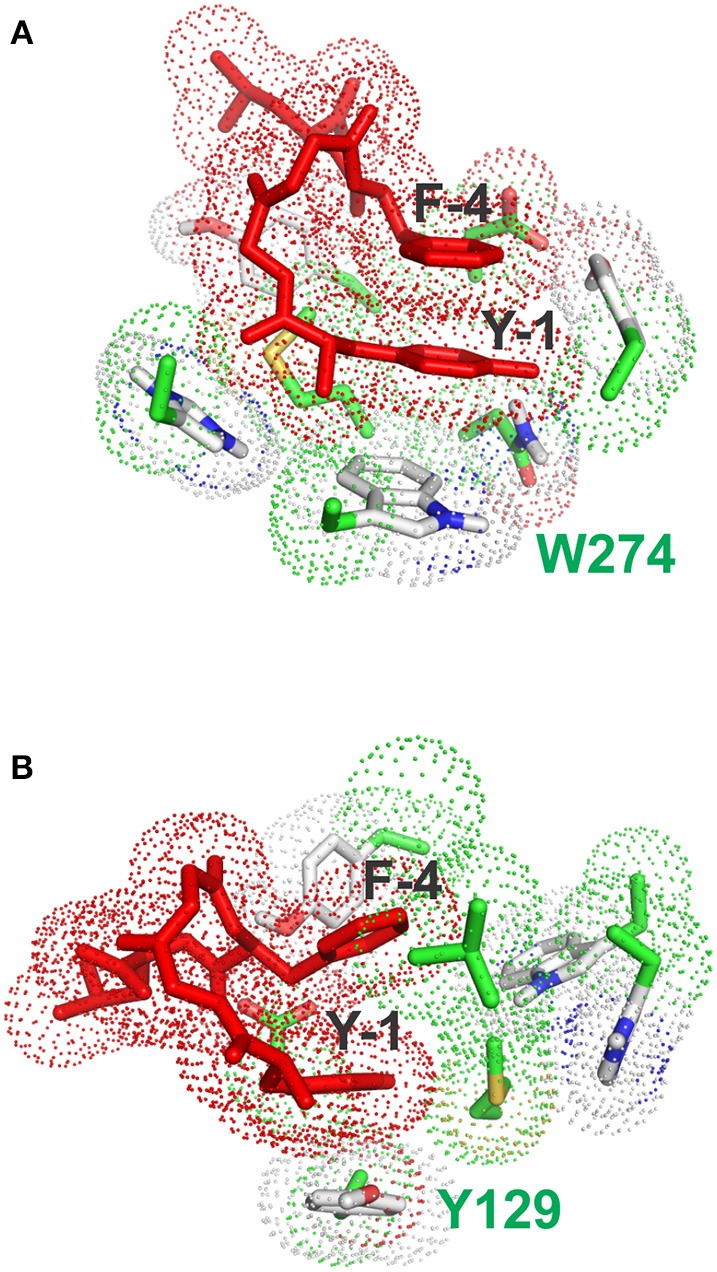
**Stacking interactions between conformers (A) and (B) and aromatic residues of the receptor. (A)** Pose with the lowest energy for conformer A. The main stacking interaction is that of the tyrosine ring of enkephalin (Y-1) with the aromatic sidechain of W274. **(B)** Pose with the lowest energy for conformer B. The main stacking interaction is that of the tyrosine ring of enkephalin (Y-1) with the aromatic sidechain of F129.

It is possible, from the analysis of Ligplot+, to identify seven residues lining the walls of the active site of the receptor that are most likely to interact with ligands. In the final stage of our docking calculations we allowed the sidechains of D128, Y129, N131, M132, W274, H278, and Y308 to move freely while the program attempted to dock rigid models of conformers A and B of enkephalin. Interestingly the results of this screening provided at least 4 poses with energy levels comparable to those of naltrindole. Figure 4A and Table 3S shows that the positions of six of these sidechains changed very little in the four poses of minimum energy. Only W274, whose indole ring flips by 180 degrees, was dislocated in a substantial way. Figure 4B shows the same view as Figure 4A, with the addition of the molecular model of naltrindole. Figures 4C,D and Figures 5A,B show the best poses for conformers A and B, respectively. The pairwise r.m.s.d's between the five side chain positions, generated by the software when accommodating the rigid ligand, range from 0.097 to 1.43, including all seven residues. Individual views are shown in Table 1S (Supplementary Material).

Interestingly, the pose with the lowest energy for conformer A is forming stacking interactions with the Tryptophan of the binding site, whereas in the crystal structure the inhibitory ring is present in that part of space, indicating a possible difference in the interaction between enkephalin and naltrindole. In B the lowest energy conformation is forming a stacking interaction with the Phenylalanine 129, as shown Figure 5B.

## Discussion

The search for the conformation of a small flexible peptide has been defined in the past as “an elusive goal” (Rose et al., 1985). This attitude was plainly justified, at the time, by the bold but inadequate means used to find bioactive conformations of flexible peptides, the “Holy Graal” of drug design. For instance, early NMR studies just looked for *the* conformation in aqueous solution (Jones et al., 1976; Roques et al., 1976), i.e., a single conformation consistent with NMR data, even if it was already clear that the interior of the (then unknown) receptor was probably far less hydrophilic than aqueous solutions (Temussi et al., 1989). Yet, there have been so many attempts to find bioactive conformations of peptides that it is interesting to check whether there is any relevant information hidden in these studies. Do 4 or 5 decades of conformational studies on the search for the bioactive conformation of small peptides, still hold any value? To answer this question we chose a paradigmatic example, i.e., the bioactive conformation of enkephalin, both because of the importance of this peptide and because the structure of its receptor has been recently determined.

We restricted our search to a small sample of conformers, the more representative found in solid state studies (Deschamps et al., 1996) and a few from the *only* solution study in which it proved possible to measure a significant number of NOEs (Amodeo et al., 1998). The result is very encouraging: some compact conformers can occupy a region of the active site of the receptor similar to that covered by naltrindole, the rigid antagonist co-crystallized with the receptor (Fenalti et al., 2014).

The conformations of the best of all conformers examined, A and B from the solution study (Amodeo et al., 1998), proved also an excellent choice as starting conformations for docking calculation inside the receptor. Even if the sidechains of residues lining the walls of the active site are held rigidly in the position of the crystal structure (pdb id 4N6H), it is easy to find low energy solutions in which crucial moieties of enkephalin end up in a position similar to those occupied by corresponding moieties of naltrindole in the crystal structure of the complex with the receptor. The two solutions are not identical, but it is not possible to discriminate between them on the basis of a simple *in silico* calculation. Even more significantly, both *rigid* conformers (A and B of the solution study) can yield low energy poses with energy score quite similar to those attained by naltrindole, provided that a small number of sidechains in the receptor wall are free to readjust. The presence of two glycine residues, viewed as a curse in conformational studies, is the key chosen by evolution to favor compact conformations in which the aromatic rings of Y1 and F4 are close in space.

Before attempting to analyze the general significance of our findings, it is in order to critically reexamine some aspects that lie at the base of our attempt. In our choice of the receptor we were obviously limited by the availability of solid state structures in which an antagonist is bound to the receptor. It is only fair to say that in general there is no warranty that any given agonist should bind in the same fashion of an antagonist. In particular a linear flexible agonist may, in principle, behave quite differently from a rigid antagonist. However, in the case of enkephalin and naltrindole, we took advantage of the extreme similarity between the rigid antagonist (naltrindole) and one of the rigid agonists (SIOM) used as a conformational sieve in the original NMR paper (Amodeo et al., 1998). The two compounds differ only by the slightly different bulk of the N substituent (a methyl in SIOM and a cyclopropyl in naltrindole). Therefore, it is very likely that, in the case of this pair, the agonist and the antagonists (both very rigid) occupy the same cavity in the receptor. This assumption implies a mechanism in antagonism that is far from proven but quite plausible. The bulkiness of the N substituent can prevent a specific motion in the receptors' helices while the bulk of the two very similar molecules reside in the same receptor cavity. Thus, it is likely that the different bulk of the N substituent can hinder a specific conformational change of the receptor without altering its global architecture.

Altogether we would like not to make too many hypotheses on the mechanism of action of opioids solely on the basis of an *in silico* study. The main goal of our paper was to show that it is possible, at least in principle, to make an intelligent use of solution studies on flexible peptides.

It is important to acknowledge that a flexible agonist can assume, inside the receptor, a conformation radically different from that of a rigid agonist. As previously mentioned, this possibility is hinted at in our work by the finding that conformers A and B seem to interact with different aromatic residues: W274 for conformer A and Y129 for conformer B. However, while it is not possible to tell in a conclusive way whether either conformer A or conformer B is *the* bioactive conformation of enkephalin, it is tempting to suggest that even inside the receptor enkephalin can interact in several related conformations, characterized by the special proximity of its aromatic rings. An ensemble of bioactive conformations would have obvious entropic advantages. It seems in order to note that a huge number of experimental and theoretical studies performed during the last 40 years failed to hint at any plausible bioactive conformation of enkephalin. We are very proud that we can now show that at least one of the conformers previously found by us in a solution study (Amodeo et al., 1998) is a likely bioactive conformation.

We can thus conclude that the goal is less elusive than it appeared many years ago. We can confidently affirm that solution studies of other bioactive peptides, however flexible, can help to understand some aspects of their bioactive conformation. The only proviso is that conformational studies be performed in media that represent a reasonable mimic of natural environments.

### Conflict of interest statement

The authors declare that the research was conducted in the absence of any commercial or financial relationships that could be construed as a potential conflict of interest.
