# Supplementary material for: The conformation of enkephalin bound to its receptor: an “elusive goal” becoming reality
Source: Front Mol Biosci. 2014 Oct 7;1:14. doi: 10.3389/fmolb.2014.00014 (PMC4428452; doi:10.3389/fmolb.2014.00014)
Supplement: Supplementary file 1 [file DataSheet1.PDF]

Supplementary Information

The conformation of enkephalin bound to its receptor: An 'elusive goal' becoming reality

Domenico Sanfelice and Piero Andrea Temussi

Table 1S. Main chain torsion angles for representative solid state conformations.

	ψ_1	ϕ_2	ψ_2	ϕ_3	ψ_3	ϕ_4	ψ_4	ϕ_5
extended	153	173	174	-174	174	-140	145	-122
single bend	130	61	23	90	-2	-120	153	-96
double bend	104	-56	-38	-54	-30	-96	-2	-73

Table 2S. Main chain torsion angles for representative solution state conformations selected using rigid conformational sieves.

	ψ_1	ϕ_2	ψ_2	ϕ_3	ψ_3	ϕ_4	ψ_4	ϕ_5
A	179	162	-50	-74	77	-90	48	-80
B	180	162	-50	-74	0	-90	48	-80
C	172	158	162	77	-8	-87	52	-138
D	173	159	166	76	-4	-85	61	60
E	-177	95	130	72	-79	-74	93	73
F	164	76	-91	-73	122	-35	-60	-78

SIDE CHAINS FLUCTUATIONS

COMBINATION	r.m.s.d (Å)
**1 - 2**	1.102
**1 - 3**	0.097
**1 - 4**	1.426
**1 - 5**	1.411
**2 - 3**	0.828
**2 - 4**	0.254
**2 - 5**	0.156
**3 - 4**	0.403
**3 - 5**	0.394
**4 - 5**	0.430

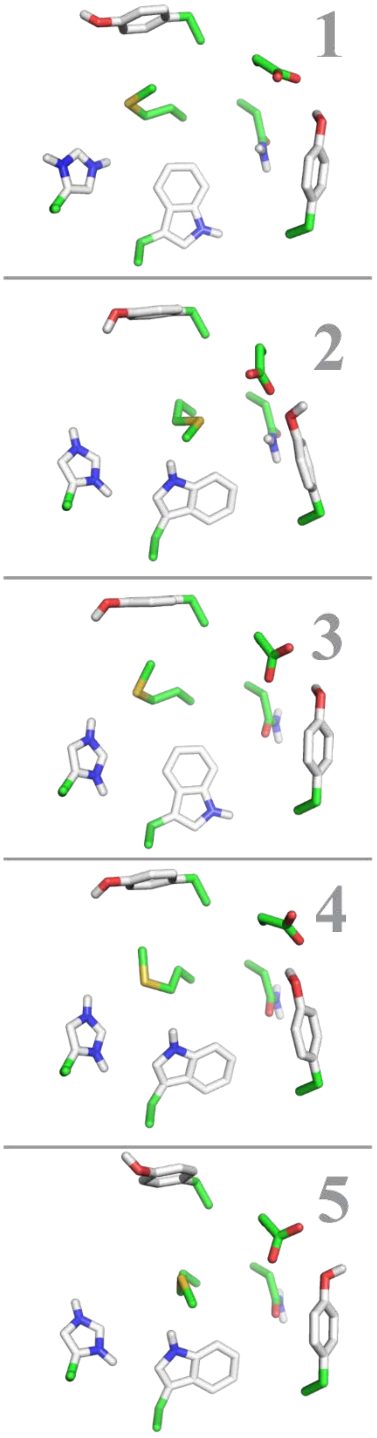

Table 3 S. Analysis of side-chain fluctuations. The side chain of 7 amino-acids belonging to the binding site were imposed flexible during calculation using AutoDock Vina. The results of the calculation lead to 5 poses that are reporter from panel 1 to panel 5. In table S.1 are reported the r.m.s.d. values calculated using Pymol (Delaglio).

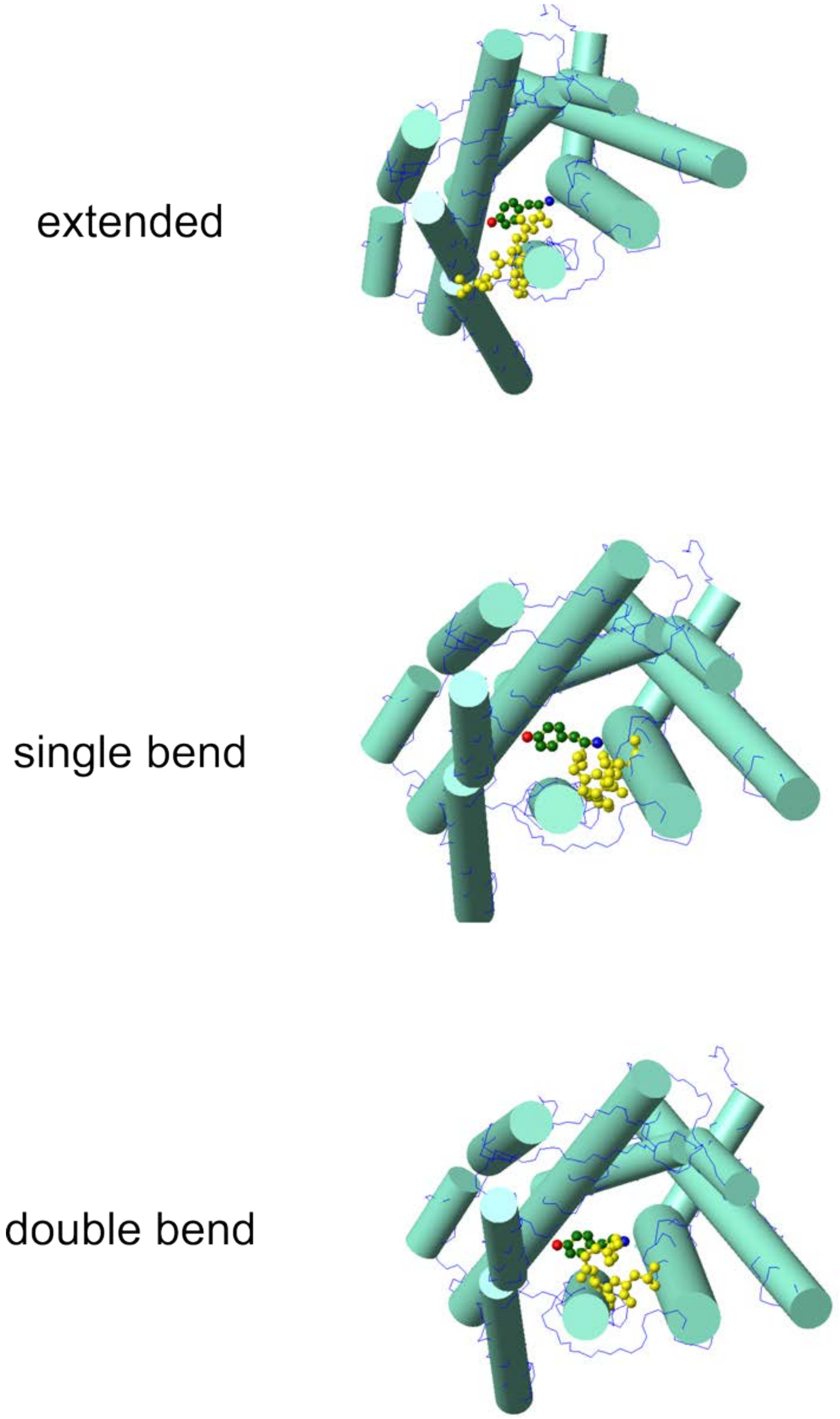

Figure 1S. Molecular models of Leu-enkephalin in the three main conformations found in the solid state, extended (xt), single bend (sb) and double bend (db) superimposed to the molecular model of naltrindole (nal) inside the receptor (pdb id 4N6H). The models were generated with MOLMOL (Koradi et al., 1996).

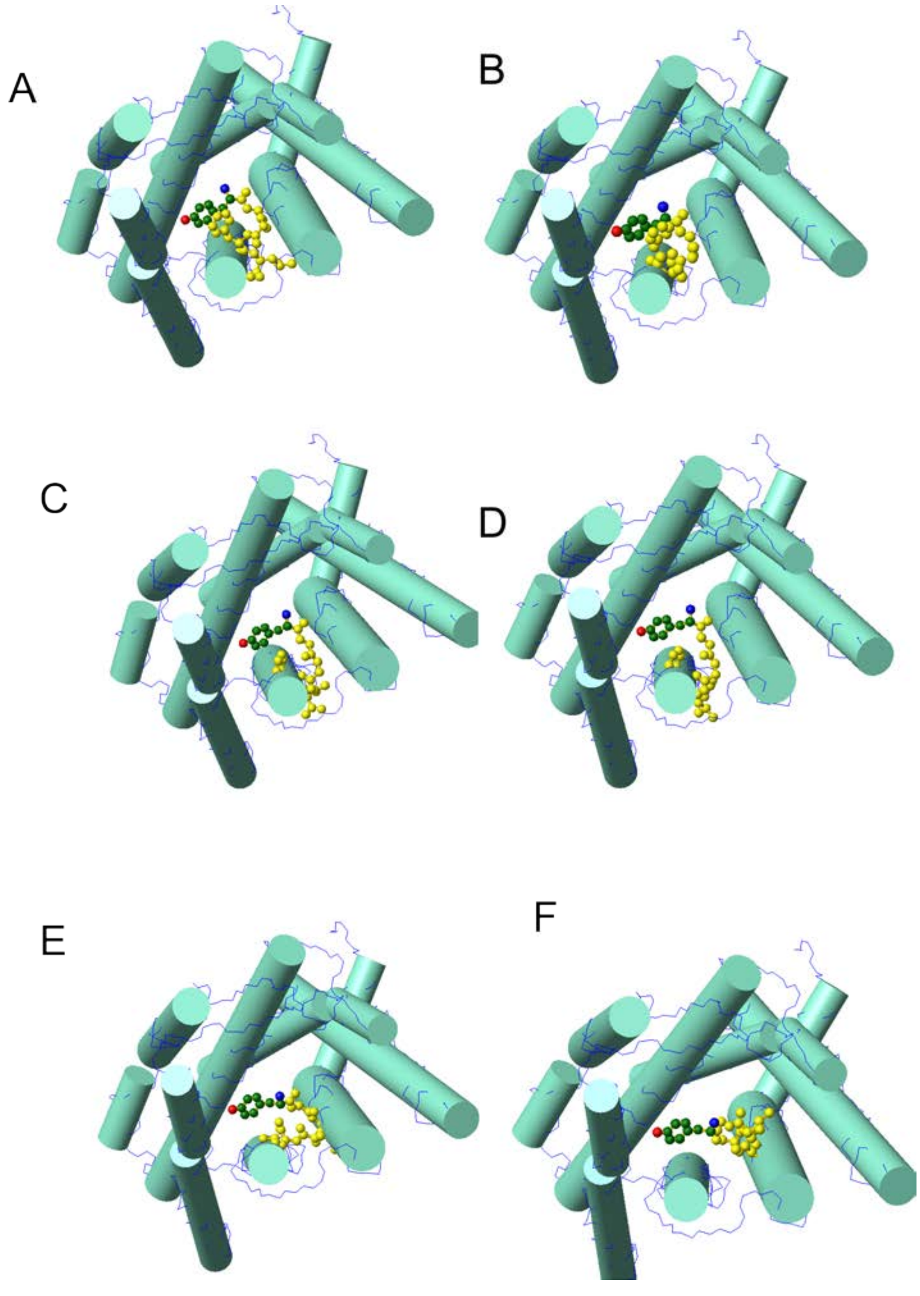

Figure 2S. Molecular models of conformers (A, B, C, D, E, F) of Leu-enkephalin found in a low temperature solution study (Amodeo et al., 1998) inside the receptor. The models were generated with MOLMOL (Koradi et al., 1996).

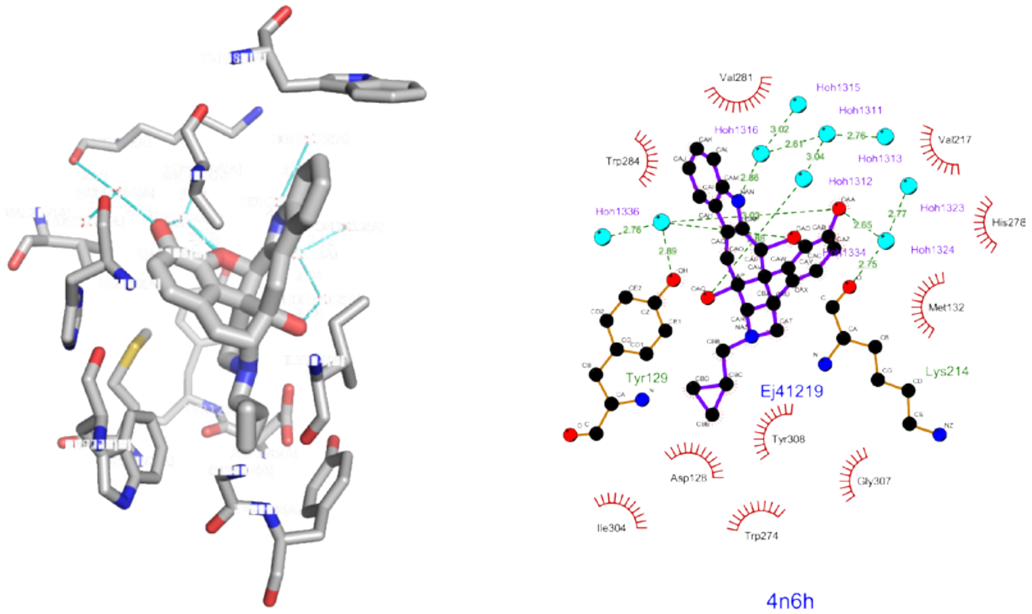

Figure 3S. Two views of the binding site of naltrindole in the pocket of 4n6h. The sidechains of the residues closest to naltrindole (D128, Y129, N131, M132, W274, H278, Y308) were chosen for the calculation with rigid enkephalin conformer and flexible receptor sidechains.

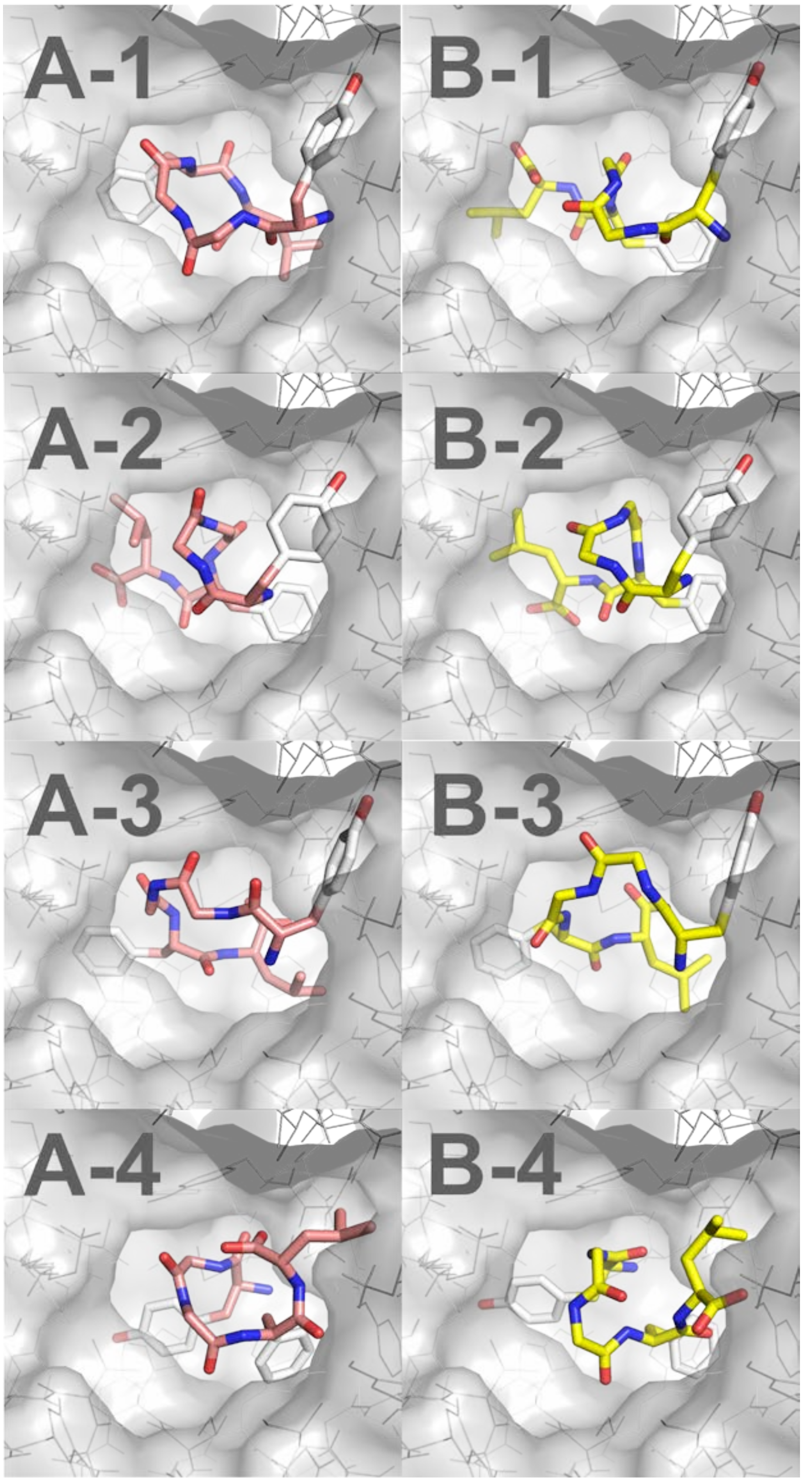

Figure 4S. Docking of flexible enkephalins. A1-A4) Molecular models generated by docking calculations starting from conformer A; all models are shown as sticks with carbon-carbon bonds colored in pink. B1-B4) Molecular models generated by docking calculations starting from conformer B; all models are shown as sticks with carbon-carbon bonds colored in yellow.
